# PI3K, p38 and JAK/STAT signalling in bronchial tissue from patients with asthma following allergen challenge

**DOI:** 10.1186/s40364-018-0128-9

**Published:** 2018-04-11

**Authors:** Thomas Southworth, Sarah Mason, Alan Bell, Isabel Ramis, Marta Calbet, Anna Domenech, Neus Prats, Montserrat Miralpeix, Dave Singh

**Affiliations:** 1 Division of Infection, Immunity & Respiratory Medicine, The Medicines Evaluation Unit, The University of Manchester, Manchester Academic Health Science Centre, Manchester University NHS Foundation Trust, Manchester, UK; 2 Almirall R&D Center, Sant Feliu de Llobregat, Barcelona, Spain; 3 The University of Manchester, 2nd Floor Education and Research Center, Wythenshawe Hospital, Southmoor Road, Manchester, M23 9LT UK

**Keywords:** Allergens, Asthma, Bronchoscopy, Immunohistochemistry, JAK-STAT signalling, Kinase, p38 mitogen-activated protein kinase, Phosphoinositide 3-kinase

## Abstract

**Background:**

Inhaled allergen challenges are often used to evaluate novel asthma treatments in early phase clinical trials. Current novel therapeutic targets in asthma include phosphoinositide 3-kinases (PI3K) delta and gamma, p38 mitogen-activated protein kinase (p38) and Janus kinase/Signal Transducer and Activator of Transcription (JAK/STAT) signalling pathways. The activation of these pathways following allergen exposure in atopic asthma patients it is not known.

**Methods:**

We collected bronchial biopsies from 11 atopic asthma patients at baseline and after allergen challenge to investigate biomarkers of PI3K, p38 MAPK and JAK/STAT activation by immunohistochemistry. Cell counts and levels of eosinophil cationic protein and interleukin-5 were also assessed in sputum and bronchoalvelar lavage.

**Results:**

Biopsies collected post-allergen had an increased percentage of epithelial cells expressing phospho-p38 (17.5 vs 25.6%, *p* = 0.04), and increased numbers of sub-epithelial cells expressing phospho-STAT5 (122.2 vs 540.6 cells/mm^2^, *p* = 0.01) and the PI3K marker phospho-ribosomal protein S6 (180.7 vs 777.3 cells/mm^2,^ *p* = 0.005). Type 2 inflammation was increased in the airways post allergen, with elevated levels of eosinophils, interleukin-5 and eosinophil cationic protein.

**Conclusions:**

Future clinical trials of novel kinase inhibitors could use the allergen challenge model in proof of concept studies, while employing these biomarkers to investigate pharmacological inhibition in the lungs.

## Background

Novel asthma treatments in early clinical development are often evaluated using the inhaled allergen challenge model [1], which enables rapid evaluation of the potential to suppress allergic inflammation before later phase studies focusing on clinical endpoints. The primary endpoint of allergen challenge studies is usually the fall in lung function caused by allergen inhalation, but biomarkers of lung inflammation can also be measured.

Intracellular kinase inhibition can potentially exert broad immunosuppressive effects on multiple cell types. Examples of kinases that have been considered as therapeutic targets in asthma are phosphoinositide 3-kinases (PI3K) delta and gamma, p38 mitogen-activated protein kinase (p38) and Janus kinase/Signal Transducer and Activator of Transcription (JAK/STAT) [2–4]. PI3Kδ and PI3Kγ are predominantly expressed in leukocytes and play a role in lymphocyte differentiation, activation, cell migration and reactive oxygen species production. In animal models of asthma, PI3K inhibition reduces inflammation and airway remodelling [5]. Furthermore, PI3Kδ expression is increased in the airways of asthma patients compared to controls [6]. p38 MAPK controls inflammatory gene transcription and translation [2] and is involved in the inflammatory responses of many different cell types to a wide range of stimuli [7–9]. In asthma patients the levels of p38 MAPK activity in the blood have been shown to increase with disease severity [10], while there is also evidence of increased alveolar macrophage and bronchial epithelial p38 MAPK activation in severe asthma [11, 12]. The JAK pathway is activated by pro-inflammatory cytokines leading to phosphorylation of STAT transcription factors [3]. JAK/STAT signalling is essential for lymphocyte differentiation and anti-viral mechanisms [13, 14]. Corticosteroids are currently the most commonly used anti-inflammatory treatment for asthma. However, in vitro studies, using human airway cells, have shown that JAK/STAT-dependent mechanisms are corticosteroid-insensitive [15, 16]. Animal models of allergic asthma show activation of PI3K, p38 MAPK and JAK/STAT pathways in the lungs [17, 18]. However, whether allergen exposure activates these pathways in humans with asthma has not been studied.

This study investigated the activation of PI3K, p38 MAPK and JAK/STAT signalling in lungs of asthma patients after allergen challenge; thus assessing the mechanistic involvement of these pathways during the human allergic response. As allergen challenge studies are often used during early clinical development of novel drugs, we also sought to identify biomarkers of PI3K, p38 MAPK and JAK/STAT activation in the lungs after allergen challenge.

## Methods

### Patients

Twelve steroid naïve atopic asthma patients were recruited; nine were male, with mean age of 45.5 and FEV_1_% predicted of 86.2%. Patient demographics and clinical characteristics can be found in Table 1. All subjects demonstrated a positive skin prick test to either cat or house dust mite extract (Aquagen® SQ, ALK Laboratories, Cophenhagen, Denmark); showed methacholine hyperresponsiveness (PC_20_ < 16 pg/ml); and previously had shown both an early (from 0 to 2 h) and late (from 4 to 10 h) asthma response following allergen challenge, defined as a decrease in FEV_1_ of > 20% and 15% respectively. Allergen challenges were performed using the Cockcroft method [19]. The study was approved by the local research ethics committee (Greater Manchester Central REC reference: 14/NW/0048) and subjects provided written informed consent.

**Table 1:** Individual Patient demographics, clinical characteristics and allergen challenge information

Number of patients	11
Age	45.5 (7.2)
Gender	Male 9 Female 2
Baseline FEV_1_ (% predicted)	101.5 (12.3)
Baseline FeNO (ppb)	34.5 (20.6)
ACQ score	1.2 (0.6)
Methacholine PC_20_	2.7 (2.2)
Allergen selected for challenge	HDM 9 CAT 2
Cumulative Allergen Concentration Administered (SQU/mL)	22,366.5 (27,672.3)
Maximum % fall in FEV_1_ during EAR	29.2 (3.9)

Results shown as mean (sd)

*Abbreviations:* *FEV*_1_ forced expiratory volume in 1 s, *FeNO* forced exhaled nitric oxide, *ppb* parts per billion, *ACQ* asthma control questionnaire, *PC*_20_ provocation concentration, *HDM* house dust mite, *EAR* early asthmatic response

### Study design

Baseline induced sputum and bronchoscopy samples were collected. Induced sputum samples were processed to obtain supernatant for protein analysis and differential cell count cytospins [20]. Bronchoscopy was performed to collect bronchoalveolar lavage (BAL) fluid for protein analysis and bronchial biopsies for immunohistochemical analysis. Bronchial biopsies were collected from right or left lower lobes. At 6 weeks after the baseline bronchoscopy, an allergen challenge was performed. This time period was to allow for patient recovery following baseline bronchoscopy sample collection. Sputum induction and bronchoscopy were carried out at 4 and 6 h post-allergen challenge respectively; this impeded FEV_1_ characterisation of the late asthma response.

Immunohistochemistry was used to assess bronchial biopsy expression of the following proteins in both the epithelium and sub-epithelium; phospho-Protein kinase B (pAKT) and phospho-Ribosomal Protein S6 (pRPS6) involved in PI3K signalling; p-p38 and phospho-Heat Shock Protein 27 (p-HSP27) involved in p38 MAPK signalling; and phospho-STATs 1, 3, 5 and 6.

Levels of IL-5 and eosinophil cationic protein (ECP) were assessed in sputum supernatants and bronchoalveolar lavage by MesoScaleDiscovery assay at SGS Cephac Europe, Saint Benoît Cedex, France. Sputum differential cell counts were assessed by Rapi-Diff stain [20].

### Cockcroft allergen challenge

Allergen (Aquagen® SQ, ALK Laboratories, Cophenhagen, Denmark) inhalations using the tidal breathing method were performed with a deVilbiss nebuliser operated by air and at a flow rate to give an output of 0.13 ml/min. The subjects wore a nose-clip and aerosol was inhaled through a mouth-piece during tidal volume breathing (V_T_). The starting concentration for the allergen was determined with results of the skin sensitivity test and the methacholine PC_20_ according to the formula published by Cockcroft et al., 1987. The starting concentration was 2 doubling doses less than predicted to cause a 20% fall in FEV_1_ during the EAR. FEV_1_ was measured twice, one minute apart, 10 min after the allergen was administered. If the FEV_1_ fell by < 15%, the next dose of allergen was administered. If the FEV_1_ fall was ≥ 15% and < 20%, FEV_1_ measurements were performed at 15 mins post allergen to see if a ≥ 20% fall was achieved. When the FEV_1_ fell by ≥ 20% or the highest dose of allergen was administered, the allergen challenge was discontinued and serial FEV_1_ measurements were performed for 4 h before performing a sputum induction. An early asthmatic response (EAR – FEV_1_ fall ≥ 20%) was assessed after 2 h.

### Methacholine challenge

Methacholine challenges, using Methacholine chloride, Stockport Pharmaceuticals, Stockport, UK, were performed if the FEV_1_ was ≥ 65% predicted. Subjects inhaled doubling concentrations of methacholine (0.03125 to 16 mg/ml) for 2 min at V_T_ with a nose clip, at 5 min intervals using the deVilbiss nebuliser until there was a fall in FEV_1_ of ≥ 20%. FEV_1_ was measured at 30 and 90 s intervals after each allergen administration and the highest value used for calculating FEV_1_ fall. Initially, a diluent control was used. Subjects with a post-diluent decrease ≥ 10% did not continue to the methacholine challenge. The provocation concentration of methacholine required to cause a fall in FEV_1_ of 20% (PC_20_) was calculated from the dose response curve.

### Serial skin prick test

The skin sensitivity test was performed to determine the allergen PC_20_ estimation for the tidal breathing challenge. Duplicate skin prick tests were performed with the fold dilutions of the same allergen (Aquagen® SQ, ALK Laboratories, Cophenhagen, Denmark) used for inhalation. The diameter of each wheal diameter was calculated from the average of the two. Cutaneous skin sensitivity was defined as the concentration producing a mean wheal diameter of 2 mm and was obtained from a plot of the log concentrations versus wheal size.

### Sputum induction

Sputum samples were obtained at the start of the study (visit 1) and at 4 h post allergen challenge at visit 3. Sputum induction was performed after administration of 200-400 μg of Salbutamol. Increasing saline concentrations (3%, 4%, 5%) was given to the patient as 3 nebulisations (Flaem Nuova EasyNeb II, Milan, Italy) each lasting for 5 min. Spirometry (Micro-loop®, Carefusion, Basingstoke, UK) was performed prior to sputum induction and after each nebulisation cycle as a safety precaution to assess the effect of inhaled saline on FEV_1_.

### Sputum processing

Selected sputum was weighed, and samples greater than 0.05 g was mixed with eight volumes of phosphate buffered saline (PBS) before centrifugation. Supenatants were collected and stored at -80 °C until required for protein analysis. Remaining cells were mixed with 0.1% DTT for 15 min. Harvested cells were re-suspended in cold PBS so that a cell count could be performed using trypan blue to assess the number of viable cells. Cytopsin slides were prepared for differential count. Cytospin preparations were air dried, fixed with methanol and stained with Rapi-diff (Triangle, Skelmersdale, UK). Each reader scored 400 cells. This was used to determine the percentage of squamous cells as a measure of sputum quality. Samples with < 50% squamous cells were scored as acceptable. The results were expressed as a percentage of the total non-squamous count, and a total cell count/g of sputum.

### Bronchoscopy and sample processing

Bronchoscopy was performed after the subjects had been sedated. BAL was collected from the right or left upper lobe. The bronchoscope was wedged in the bronchus and a maximum of 4 × 60 ml aliquots of pre-warmed sterile 0.9% saline solution were instilled. The aspirated fluid was stored on ice before filtration (100 μm filter, Becton Dickenson, Oxford, UK). The filtrate was centrifuged (400 *g*/10 min at 4 °C) and the BAL fluid was stored at -80 °C in 1 ml aliquots for biomarker analysis. The cell pellet resuspended in PBS and viable cell counts were determined by trypan blue exclusion (Neubauer hemocytometer), and the suspension adjusted to 0.5 × 10^6^ BAL cells/ml. Cytospins for differential cell counts were made using 100ul of cell suspension. Bronchial biopsies were collected from the right or left lower lobes.

### Immunohistochemistry

Bronchial biopsies were fixed overnight in 10% neutral buffered formalin (CellPath, Powys, UK), and embedded in paraffin wax (Surgipath, Milton Keynes, UK). 3 μm tissue sections were cut and lifted onto a polysine coated glass slides, before dewaxing in xylene and rehydrated with decreasing concentrations of alcohol. Following heat induced epitope retrieval (20 min in microwave at 800 W); tissues were stained overnight with primary antibody, before detection with biotinylated anti-rabbit IgG secondary antibody (Vector Laboratories, Peterborough, UK). Staining was visualised using avidin biotin peroxidase complex (Vector Laboratories). All sections were counterstained with Gill’s II haematoxylin (Leica, Milton Keynes, UK). Quantification was performed using the ImagePro Plus 6.0 software. All antibodies were purchased from Cell Signalling Technology, Davers, USA, except for pSTAT6 (Life Technology, Paisley, UK): pAKT product #9271; pRPS6 product #5364; p-p38 product #9211; p-HSP27 product #2401; phospho-STAT 1, 3, 5 and 6 product codes 9167, 9145, 9359 and 700,247, respectively. Sub-epithelial cell counts for neutrophils (neutrophil elastase), eosinophils (Luna stain), CD4^+^ and CD8^+^ cells were performed as previously described [21]. Positive staining was confirmed by use of two negative controls: (i) exclusion of primary antibody and (ii) use of isotype control antibody.

### Differential cell count

Sputum and BAL cell cytospins were fixed in methanol for 10 min and left to air dry, before being stained with Rapi-Diff (GCC Diagnostics) using the following method: (1) stain slide for 2 min in solution A; (2) wash slide in distilled water; (3) stain slide for 2 min in solution B; (4) wash slide under tap water. Slides are then mounted using DPX and cover slipped. 400 non-squamous cells were counted per slide. Neutrophils, macrophages, eosinophils, lymphocytes and bronchial epithelial cells differentiated based on morphology and staining.

### Statistical analysis

Data distribution was assessed by Kolmogorov-Smirnov test. Data with non-parametric distribution are highlighted in figures with an asterisk. Changes in cell counts and protein levels pre- and post-allergen challenge were analysed by two-sided paired T-tests or Wilcoxon tests, where appropriate. All analysis was carried out using Prism 7.0. software (Graphpad, La Jolla, California, USA). A *p*-value of less than 0.05 was deemed significant.

## Results

One subject was withdrawn after not tolerating the baseline bronchoscopy, with 11 completing the study. All subjects demonstrated an early asthma response with a fall in FEV_1_ of > 20% within 20 min of allergen inhalation, with a mean maximum fall of 29.2%.

Paired baseline and post-allergen sputum samples were successfully collected from 10 of the 11 patients. Allergen challenge increased sputum eosinophil percentage (from mean 1.1% to 3.8%; *p* = 0.019) and cell count (from median 0.01 to 0.12 × 10^6^ cells/g sputum; *p* = 0.008). Neutrophil percentage and cell counts also increased (from mean 37.9 to 63.4%, *p* = 0.004, and from 0.8 to 2.0 × 10^6^ median neutrophils/g sputum: *p* = 0.05). The macrophage percentage decreased (from mean 49.9 to 28.6%, *p* = 0.02) after allergen challenge. There were no changes in sputum lymphocyte or epithelial cell numbers.

**Fig. 1:**
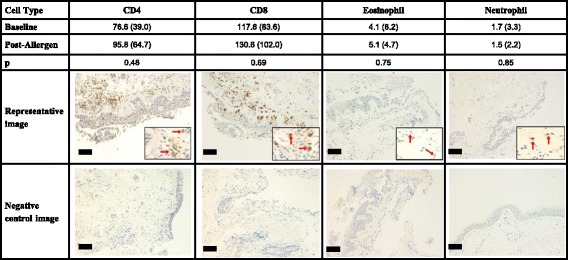
Inflammatory cell counts in sub-epithelial bronchial tissue at baseline and post-allergen (*n* = 11). Bronchial biopsies were collected from 11 asthma patients at baseline and 6 h post allergen challenge, with CD4, CD8 and neutrophil numbers being assessed by immunohistochemistry and eosinophils with Luna staining. Data presented as mean (sd). Comparisons between Baseline and Post-allergen by paired T-test. Images of staining are at × 200 magnification; Black bars represent 100 μm. Magnified images and red arrows illustrate positive staining. Negative control images represent isotype controls results for CD4, CD8 and neutrophil elastase, and non-Luna staining for eosinophils

Type 2 inflammation was increased by allergen challenge; sputum supernatant ECP (57 vs 169 ng/ml; *p* = 0.049) and IL-5 (0.9 vs 19.9 pg/ml; *p* = 0.016) levels were higher after allergen challenge. BAL ECP levels were increased by allergen challenge (1.9 vs 3.2 ng/ml; *p* = 0.014). The higher levels measured in sputum supernatant was probably due to the know dilution effect of BAL, but the median fold change in ECP was similar in sputum supernatant and BAL (2.7-fold and 2.0-fold, respectively). Levels of IL-5 in BAL were undetectable, both at baseline and post allergen. Allergen exposure had no effect on neutrophil, macrophage, eosinophil and lymphocyte cell numbers in bronchial tissue (Fig. 1).

**Fig. 2:**
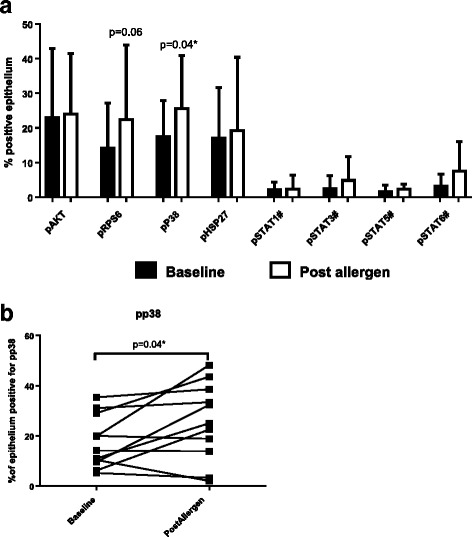
Levels of PI3K, p38 and JAK/STAT markers in bronchial epithelium at baseline and post allergen (*n* = 11): Bronchial biopsies were collected from 11 asthma patients at baseline and 6 h post allergen challenge, with protein expression analysed in the epithelium (**a**) by immunohistochemistry. Data presented as mean values with standard deviation. Individual patient’s data for the statistically significant changes in pp38 are shown in (**b**). Comparisons between baseline and post allergen were by paired T-tests or Wilcoxon tests (#), as appropriate *p < 0.05

**Fig. 3:**
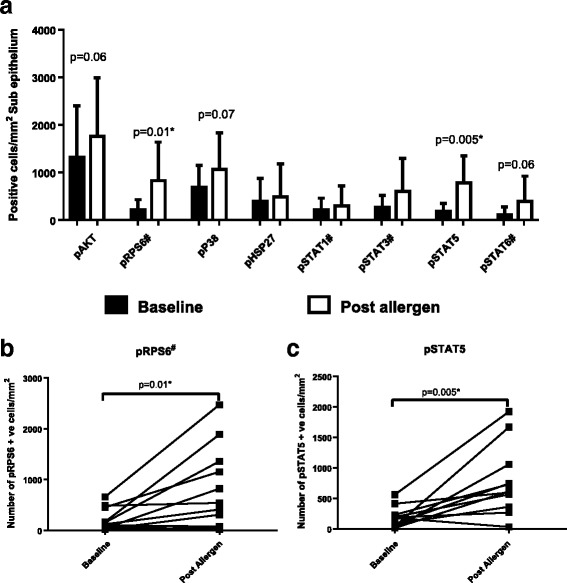
Levels of PI3K, p38 and JAK/STAT markers in bronchial subepithelium at baseline and post allergen (*n* = 11): Bronchial biopsies were collected from 11 asthma patients at baseline and 6 h post allergen challenge, with protein expression analysed in the epithelium (**a**) by immunohistochemistry. Data presented as mean values with standard deviation. Individual patient’s data for the statistically significant changes in pRPS6 and pSTAT5 are shown in (**b**) and (**c**), respectively. Comparisons between baseline and post allergen were by paired T-tests or Wilcoxon tests (#), as appropriate *p < 0.05

**Fig. 4:**
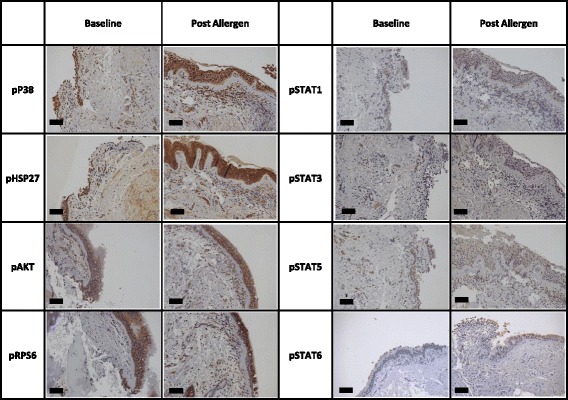
Representative images of cell signalling markers in bronchial biopsy tissue. Bronchial tissue collect pre and post allergen challenge were stained by immunohistochemistry for phosphorylated cell signalling proteins. All representative images are from the same patient. Black bars represent 100 μm

In bronchial epithelium there was a significant increase in the percentage of cells expressing p-p38 post-allergen (p = 0.04; Fig. 2). The percentage of pRPS6 positive epithelial cells was also increased, but this did not reach significance (*p* = 0.06). Numbers of sub-epithelial cells expressing pRPS6 and pSTAT5 were increased post-allergen (*p* = 0.01 and *p* = 0.005 respectively, Fig. 3). Numbers of pAKT, p-p38 and pSTAT6 positive sub-epithelial cells were also increased, but these did not reach significance (*p* = 0.06, *p* = 0.07 and *p* = 0.06 respectively). Other proteins analysed in epithelium (pHSP27, pAKT, pSTATs 1, 3, 5 and 6) or sub-epithelium (pHSP27, pSTATs 1 and 3) by immunohistochemistry did not show any change after allergen challenge. Representative images of bronchial tissue staining are shown in Fig. 4.

## Discussion

There was an increase in p-p38 MAPK expression in the bronchial epithelia after allergen challenge, while in the sub-epithelium there was evidence of PI3K activation (pRPS6) and JAK/STAT activation (pSTAT5). These observations support involvement of these kinase pathways in the allergic response, but with different roles in the epithelium compared to the sub-epithelium.

Phospho-p38 MAPK expression is increased in the bronchial epithelium of patients with severe asthma [12, 22]. We now show p38 MAPK activation at the same anatomical location after allergen challenge. Overall, these findings suggest that epithelial p38 MAPK activation contributes to the inflammatory cascade in asthma caused by environmental triggers, such as allergen exposure. In vitro studies using human bronchial epithelial cells have shown that the house dust mite antigen, DerP1, induces p38-dependent cell apoptosis and intracellular oxidative stress [5], while in mice p38 is essential for allergen induced epithelial production of IL-25 and thymic stromal lymphopoietin (TSLP), which are both initiators of the Type-2 allergic response in asthma [10].

There is evidence that PI3K activation is increased in severe asthma [23]. PI3Kδ has a central role in T-cell activation [4]. The allergic response involves T-helper 2 cell activation, suggesting that PI3K activation could occur after allergen challenge. RPS6 is a terminal signalling protein of the PI3K/AKT/mTOR pathway, involved in protein synthesis and cell cycle progression [24]. Increased pRPS6 levels in the sub-epithelium suggest PI3K activation by allergen challenge, perhaps due to increased T-cell activity [25], although we did not specifically evaluate this.

Increased pSTAT5 expression suggests JAK/STAT pathway involvement in sub-epithelial leukocyte activation after allergen challenge. In murine lungs, STAT5 in dendritic cells is essential for TSLP-induced Th2 responses following allergen exposure [26], while STAT5 is also involved in mast cell functions, such as expression of the high affinity IgE receptor [27]. Other STAT proteins did not display changes that reached statistical significance.

The increased sub-epithelial expression of pSTAT5 and pRPS6 after allergen challenge was not due to an increase in cell numbers, as there were no increases in sub-epithelial leukocyte numbers. There are inconsistent reports of changes in leukocyte numbers in the bronchial mucosa after allergen challenge, probably due in part to differences in the time-point of sampling [28, 29]. This contrasts with the increase in luminal eosinophilic inflammation after allergen challenge, which we and others have observed [30]. Although we used a limited sample size, it was sufficiently large to observe the expected upregulation of airway luminal eosinophilic inflammation, evidenced by increased eosinophil numbers as well as IL-5 and ECP levels [31].

The expression of protein phosphorylation can be very transient e.g. pAKT expression can be short-lived in contrast to pRPS6 expression [32]. This may explain why we did not observe positive results for all the biomarkers within a kinase pathway e.g. pRPS6 expression, but not pAKT, in the sub-epithelium. We were limited in the quantity of tissue available to perform validation experiments using a different technique, such as Western blotting. Nevertheless, our results demonstrate biomarkers within these kinase pathways that show the most promise for application during the study of allergic responses in human lungs.

## Conclusions

This study presents candidate biomarkers of kinase activation during the asthmatic allergic response. Future clinical trials of novel kinase inhibitors could consider using the allergen challenge model in proof of concept studies, while employing these biomarkers to understand the pharmacological effects of kinase inhibition in the lungs.

## Abbreviations

ACQ: Asthma control questionnaire
BAL: Bronchoalveolar lavage
DTT: Dithiothreitol
EAR: Early asthma response
ECP: Eosinophil cationic protein
FEV_1_: forced expiratory volume in one second
Ig: Immunoglobulin
IL: Interleukin
JAK: Janus Kinase pAKT phosphorylated AKT
PBS: Phosphate buffered saline
PC_20_: provocation concentration producing a 20% fall in FEV_1_
pHSP27: phosphorylated heat shock protein 27
PI3K: Phosphoinositide 3-kinase.
p-p38 MAPK: phosphorylated p38 mitogen-activated protein kinase
pRPS6: phosphorylated ribosomal protein S6
pSTAT: phosphorylated Signal Transducer and Activator of Transcription
TSLP: Thymic stromal lymphopoietin
V_T_: Tidal volume breathing
